# Foundations of operator state monitoring system development in aviation

**DOI:** 10.3389/fphys.2026.1887848

**Published:** 2026-10-16

**Authors:** Katharine Woodruff, Andrew Thatcher, Güliz Tokadlı, Tyler Gandy, Nicholas Jones

**Affiliations:** 1 Advanced Aerospace Technology, Applied Research and Technology, Collins Aerospace, Cedar Rapids, IA, United States; 2 Human Factors, Product Safety, Collins Aerospace, Cedar Rapids, IA, United States; 3 Displays, Computing & Network, Avionics, Collins Aerospace, Cedar Rapids, IA, United States

**Keywords:** adaptive cockpit systems, alerts and interventions, human performance, human reliability assessment, operator state monitoring, pilot state assessment

## Abstract

The role and responsibilities of pilots have been evolving as advanced automation systems are being integrated for flight operations. Operator state monitoring (OSM) helps ensure safety and improve performance by detecting and predicting degraded operator state during operations. Despite growing interest in OSM, no unified framework exists that integrates impairment definitions, sensor criteria, and interface design requirements. This work is presented as a conceptual analysis that establishes a foundational framework for operator state monitoring system (OSMS) development, addressing what an OSMS should monitor, what types of operator state changes should be detected, the characteristics of sensing and communication needed to support these functions and the operational considerations that should inform future development. A literature review establishes potential impairment and incapacitation events for pilots, identifies relevant physiological features to support detection of these events, and assess the possible impact of such events on flight operations to inform a system response. Existing work concentrates on military and automotive contexts and on workload, fatigue, and drowsiness rather than the acute-event end of the impairment continuum; the framework developed here spans the normal to impaired to incapacitated range required for in-flight operations. OSMSs should be developed based on a multi-modal data approach consisting of physiological features, pilot performance and activity, aircraft state and environmental data, as well as contain a means for pilots to self-report input. Heart, breathing, and behavioral features are affected across the widest range of events, making them broad-coverage candidates for sensor selection, though breadth of coverage is distinct from diagnostic specificity and no single feature is treated as a standalone indicator. The remaining recommendations address the engineering of sensing and processing for the flight deck. Design guidance for the alert and notification interface is presented alongside the recommendations. As OSMSs mature, this framework can serve as a critical reference for developing robust, reliable, and widely accepted solutions in aviation.

## 1 Introduction

The role and responsibilities of pilots have been evolving as advanced automation systems are being integrated for flight operations. Increasing automation reduces active pilot workload, leading to potential skill degradation over time (Volz et al., 2016). Pilots need specific training and/or selective flight tasks to maintain their skill level during the flights. Operator State Monitoring (OSM) helps ensure safety and improve performance by detecting and predicting degraded operator state during operations. Today, crew members can monitor each other, report cases of degraded pilot states, and support one another. However, single pilot operations do not have peer-to-peer monitoring support to mitigate any degraded pilot state. To provide a peer-to-peer, support-like solution, an Operator State Monitoring System (OSMS) can detect and report changes in pilot state. This can serve as a feedback mechanism that helps a pilot take action to recover themselves, if possible, during the flight. If recovery of the degraded pilot is not possible, emergency response mechanisms can be triggered to maintain flight safety as well as the pilot and passenger safety.

This work is presented as a conceptual analysis that aims to establish a foundational framework for OSMS development, synthesizing and critically integrating relevant literature, existing practices, and considerations related to OSM to develop conceptual requirements. The primary operational scenario for this framework is single-pilot operations, where the loss of peer monitoring removes the detection pathway that currently identifies most in-flight impairment. The framework is also intended to generalize to two-pilot and reduced-crew operations, where an OSMS supplements rather than replaces cross-checking. The recommendations within are stated at a level that supports adaptation across commercial, military, and training contexts, with domain-specific constraints noted where they diverge. The proposed framework addresses:

- what an OSMS should monitor,
- what types of operator-state changes should be detected,
- the characteristics of sensing and communication needed to support these functions, and
- the operational considerations that should inform future OSMS development.

The recommendations are therefore intended to provide a basis for engineering trade-off studies for technology selection and system design. It is also important to note that this proposed framework is not intended to diagnose chronic medical conditions or pre-flight fitness issues. Rather, this research intends to address a critical gap in aviation safety: acute, in-flight operator state degradations that may not be reliably identified through medical certification or pre-flight self-assessment procedures.

## 2 Related work

Pilot incapacitation is a rare event. A 2018 FAA study found that inflight medical incapacitations affected fewer than 0.1% of the U.S. airline pilots over a 21-year period (1995-2015), with an estimated rate of 0.058 events per 100,000 flight hours (DeJohn et al., 2018). While rare, when pilot incapacitation does occur, it can pose a risk to operations and have direct consequences such as injuries, fatalities, and loss of aircraft, if not addressed. Moreover, there are likely instances of pilot impairment that occur during operations that are not significant enough to report or are resolved by the second pilot. OSMS is a solution that continuously monitors the human operator during the mission and notifies them regarding their state for possible predicted degradation (Vogl et al., 2025). No certification standards or industry guidelines currently exist for OSM in aviation. Some previous research in this space has been focused primarily on military aviation (Shaw and Harrell, 2023; Warfighter Performance Group, 2024). Many of the principles of OSM are shared between military and commercial applications, but differences such as task demands and environmental conditions (G-forces, oxygen supply, vibrations) change the typical manifestations of incapacitation. The acceptable types of sensors and system architectures also vary based on these factors, with dynamic, fast-paced military environments demanding a higher level of hardiness and motion robustness. Other research of OSM more pertinent to commercial aviation include discussions of the implications of OSM in single pilot operations (Brokaw et al., 2022) and reviews of adjacent fields like automotive drivers (Teichmann et al., 2024; Babur et al., 2025; Petrova et al., 2025). While there are many similarities in OSM between aviation and automotive applications, there are key differences in areas such as operator technical competency requirements, medical standards, and the type/timeline of system response to a suspected incapacitation.

While this body of work establishes the feasibility of OSM, three gaps remain for flight deck deployment. First, existing work concentrates on workload, fatigue, and drowsiness rather than the acute-event end of the impairment continuum; no reviewed approach spans the full normal-impaired-incapacitated range that in-flight operations require. Second, the military and automotive contexts that dominate the literature have different sensing constraints than the commercial flight deck, so what transfers is limited. Third, airworthiness constraints, multi-modal fusion, and the alerting interface are treated as downstream implementation concerns rather than as first-order design drivers. The framework developed in the remainder of this paper addresses these gaps, connecting the design recommendations in Section 3.3 to the event taxonomy in Section 2.3 and the detection framework in Section 3.1.

### 2.1 Existing preventive methods

There are already medical and technical competency evaluations for all levels of aviation, escalating in rigor from general aviation to commercial to military. Some medical conditions are completely disqualifying from medical certification as the risk of inflight incapacitation is unacceptably high. A common rule of thumb used in practice is the 1% rule, which states that in order to achieve an acceptable level of safety (ALoS) a pilot must not have greater than a 1% risk of medical incapacitation per year (Greenhaw and Tvaryanas, 2023). Examples of medical conditions that are expected to be detected at a medical examination (and thus out of scope for our present work) are epilepsy or history of a heart attack.

The second line of defense for pilot health safety/assurance is the Illness, Medication, Stress, Alcohol, Fatigue, Eating (IM SAFE) checklist. Prior to flight, pilots perform the IM SAFE checklist to assess their personal fitness and readiness to fly (Federal Aviation Administration, 2023). The checklist consists of six questions regarding pilot state (see **Table 1**).

**Table 1:** IM SAFE checklist [adapted from (Bloomstone, 2015)].

I	(Illness)	Am I ill? [Am I well]?
Factor		Self-assessment question
M	(Medication)	Am I taking any new medications or over-the-counter medications that may affect my performance?
S	(Stress)	Am I suffering any psychological or emotional factors that might affect my performance?
A	(Alcohol)	Have I recently used alcohol and/or drugs?
F	(Fatigue)	Am I sufficiently rested?
E	(Eating)	Am I sufficiently fed, watered, and relieved?

Collectively, medical certification, IM SAFE, and existing cockpit monitoring form safety barriers, but none reliably detects degradations that develop acutely in flight — hypoxia, spatial disorientation, sudden cardiac events, or contaminated bleed-air exposure. This gap motivates the development of an OSMS capable of real-time monitoring and early detection of pilot state changes during flight.

### 2.2 Definitions: pilot impairment and incapacitation events

A fundamental barrier to OSMS development is definitional inconsistency, creating ambiguity in how systems should classify and respond to pilot state degradation. The Federal Aviation Administration (FAA) provides distinct definitions of incapacitation and impairment. The FAA defines inflight medical incapacitation as “*a condition in which a flight crew member could not perform any flight duties*”, and impairment was defined as “*a condition in which a crewmember could perform limited flight duties, even though performance may have been degraded*” (DeJohn et al., 2018). On the other hand, the International Civil Aviation Organization (ICAO) defines pilot incapacitation as “*any physiological or psychological state or situation that adversely affects performance*” (ICAO, 2024). These definitions differ in scope and granularity which can leave ambiguity in how to categorize events and their performance indicators. The FAA distinguishes impairment from incapacitation as separate categories, while ICAO offers a single broad definition.

This work proposes three distinct pilot states: normal, impaired, and incapacitated. In the normal state, the pilot is fully capable of performing all flight duties without degradation. In the impaired state, the pilot’s physiological or cognitive function is degraded such that some flight duties are limited, but the pilot retains partial capability and, in many cases, the potential for recovery. In the incapacitated state, the pilot cannot perform any flight duties and recovery is unlikely. Impairment and incapacitation represent points along a continuum of degradation rather than independent conditions, with impairment typically preceding incapacitation when an event progresses untreated. This framing underpins the detection logic, sensor selection, and response requirements developed throughout the remainder of this paper. Therefore, the definitions for impairment and incapacitation are:

- A pilot impairment event is any physiological or psychological state or situation that manifests in-flight, thereby limiting a pilot’s ability to carry out their normal duties (ICAO, 2024).
- A pilot incapacitation event is any physiological or psychological state or situation that manifests in-flight, thereby preventing the pilot from performing any flight duties (Larcher and DeJohn, 2021; ICAO, 2024).

These impairment and incapacitation events occur during flight operations when they cannot be reliably detected prior to flight through preexisting conditions or risk factors, working history, and/or pilot’s self-assessment. The events are of concern whenever the flight is brought below an ALoS. Some of these events may not immediately affect flight performance (e.g., if in autopilot), but if not addressed they eventually will.

OSM should model and detect pilot state changes using both physiological event-related and performance-related indicators. These indicators may appear suddenly, where the pilot cannot self-report the occurrence prior to an incapacitation. In other cases, a slow or low-amplitude onset means the pilot may not notice or be able to judge the severity of their impairment. It is important to monitor both classes of indicators as some degraded pilot states may impact performance before a significant physiological change occurs, while other degradations have a window of clear physiological changes prior to performance decline. The OSMSs may yield insight into the level of severity and potential for recovery from the impairment. With these definitions established, the next step is identifying which in-flight events an OSMS must detect and how those events manifest physiologically, behaviorally, and operationally.

### 2.3 Summary of selected pilot state degradation events

The framework above is only actionable if the potential events are identified. A structured narrative literature review was performed in three layers to support three objectives: 1) identify potential impairment and incapacitation events for pilots, 2) identify relevant physiological features and metrics to support detection of these events, and 3) assess the possible impact of such events for flight operations to inform a system response to address it. First, authoritative review and guidance documents from aviation regulatory and research bodies were used to identify the key set of pilot impairment and incapacitation events relevant to flight operations, including the FAA (DeJohn et al., 2018), ICAO (ICAO, 2024), NTSB (Parenteau et al., 2023), and EASA (EASA, 2025). Second, literature detailing the manifestation of each event was collected, to help determine most likely detection method. Lastly, empirical evidence from the literature were collected for selected events to identify the behavior of physiological signals under each condition in relevant aircraft environments. Key terms included pilot incapacitation, aerospace medical, inflight impairment, and event names. All searches were conducted through PubMed, Google Scholar, and university library databases. The search was not exhaustive; papers were retained if they contributed to one of the three objectives and were excluded where they addressed only chronic conditions detectable at medical certification or reported no manifestation or physiological signal data. This process retained 102 documents. The subsequent sections summarize the selected pilot state degradation events, definitions, and detection methods.

An event was included if it manifests in-flight, produces changes detectable through physiological, behavioral, or performance signals, and is not reliably screened out by medical certifications or pre-flight assessment. Prior literature addresses these events in isolation across medical, military, and automotive domains. This review consolidates them into a single aviation-relevant framework. Categories sit at different levels of clinical abstraction: some describe a physiological condition (hypoxia), others an operator state (fatigue, stress) or a perceptual failure (spatial disorientation), because the organizing question is what an OSMS can observe, not what a clinician would diagnose:

- Cardiac events are any events or conditions related to the cardiovascular system (Ye, 2018). Primary symptoms include dizziness, faintness, weakness, tachycardia, chest pain, dyspnea, nausea, headache, vomiting, affected vision, coughing, shortness of breath, paralysis, edema, pallor, loss of consciousness, apnea, cardiopulmonary arrest, abdominal pain, sweating, cyanosis, numbness, and speech impairment (Goff et al., 1998). Other relevant symptoms, which might not be directly associated with cardiac events, are cognitive function decline, confusion, impaired judgement, irritability, altered consciousness, and anxiety (Selker et al., 1997; Leto and Feola, 2014).
- Fatigue is the feeling of tiredness and/or a lack of energy which can degrade the operator’s ability to perform duties (Bendak and Rashid, 2020). Operator’s fatigue state can be synonymously used with operator drowsiness (Murugan et al., 2020). Primary symptoms are sleepiness and weakness. The other relevant symptoms can be cognitive function decline, impaired judgement, and slower reaction time.
- Respiratory events negatively affect the breathing or airflow system. Primary symptoms are dizziness, faintness, fatigue, headache, nausea, pallor, shortness of breath, sweating, thirst, increased weakness, fever, coughing, chest pain, cyanosis, tachycardia, apnea, cardiopulmonary arrest, and sleepiness (Awtry et al., 2006).
- Hypoxia is defined as the inadequate level of oxygen within the cells (Richard, 1996; Amini et al., 2012). Primary symptoms are shortness of breath, headache, dizziness, nausea, dimmed vision, double vision, blurry vision, sleepiness, faintness, weakness, and stupor (Temme et al., 2010; Seech et al., 2020).
- Spatial disorientation can be defined as a pilot’s confusion about their aircraft’slocation, movement, or posture relative to the Earth’s surface and gravity (Spatial Disorientation | SKYbrary Aviation Safety, n.d.). Primary symptoms are erroneous flight control inputs or behaviors in addition to increased heart rate (HR), increased skin conductance, and decreased theta oscillations in the brain (Geva et al., 2025).
- Stress is comprised of three elements: a state of heightened excitability and arousal, perceivedaversive experiences, and experiences perceived to be beyond one’s control or predictability (Masi et al., 2023; Stress | SKYbrary Aviation Safety, n.d.).

These events differ in their onset profile, ranging from sudden incapacitation (e.g., cardiac arrest) to gradual degradation (e.g., fatigue). An OSMS must therefore detect different degradation onsets. The next section presents a detection framework.

## 3 Operator state detection system framework for pilots

Multi-modal OSM is not currently part of routine aviation operations; therefore, pilot impairment and incapacitation events are only viewed through the lens of poor performance and detected mainly by self-report. One of several safety barriers for future flight operations which would integrate increasingly autonomous systems is the lack of current OSM practices (two-pilot cross checking) to maintain an ALoS. This research hypothesizes that pilot state changes, based on the associated events, can be most effectively detected through a multi-modal approach which consists of pilot self-report, pilot performance metrics, physiological signals, aircraft state, and environmental factors. While a hazardous change to the aircraft state is ultimately what leads to aviation accidents, there are often precipitating factors that occur well before a flight is truly at risk. By monitoring these risk factors in addition to the actual flight state, there is an opportunity for faster detection and reduced accident rates. This section provides an overview of detection methods and system response as a part of the proposed framework, followed by recommendations and guidance for designing OSMS for pilots.

### 3.1 OSMS detection methods

Three detection methods are examined in this section: self-report, physiological/behavioral monitoring, and task performance/flight path monitoring. This multi-modal approach draws inspiration from the Human Factors Analysis and Classification System (HFACS), developed by the United States Navy as a structured tool for human factors error analysis (U.S. Department of Defense, 2022). HFACS models accidents as the result of a chain of broken safety barriers, organizational influences, supervisory factors, preconditions to unsafe acts, and the unsafe acts themselves, each of which provides an opportunity to prevent a hazard from progressing. Under this framework, pilot impairment and incapacitation are classified as preconditions to unsafe acts: physiological or cognitive conditions that degrade the pilot’s ability to perform safely. Detection and response at this stage represent the last barrier before the precondition manifests as an unsafe act and, potentially, an accident. This framing motivates the design goal for an OSMS: to detect impairment as early as possible within the precondition window, ideally before it produces observable degradation in flight performance.

The research-identified impairing or incapacitating events were assessed using the decision flow in **Figure 1**, which categorizes the event based on the earliest possible barrier capable of detecting it. The process starts with the question, “Can the precondition or risk of unsafe act be reliably detected prior to flight?”. If yes, the event can be expected to be detectable during the medical certification process (for longer-term conditions) or the pre-flight IM SAFE checklist. A challenging factor in this step is the difference between what is detectable by the letter of the law and what is detectable in practice: where pilots are uncomfortable disclosing mental health conditions or pre-flight fatigue/illness, relevant events cannot be reliably detected. The next question, “Does the precondition manifest or unsafe act occur in-flight?”, refers to whether the event happens in flight. While some events are coupled with the flight environment (hypoxia, task saturation), others are a function of the pilot’s health, making it a matter of probability whether the onset occurs in-flight. The remainder of the diagram fleshes out how an in-flight event would be detected. If it is always self-recognizable, it is distinguished by if it would first be recognized through its impact on health status (such as onset of a headache) or flight status (such as realizing a checklist item was missed). From there, it is further stratified through the pilot’s ability to self-report during the time of manifestation and if it is an immediate risk to operations. If the unsafe act or precondition is not always self-recognizable, then it becomes relevant to identify how automation would first recognize it, again either through a change aircraft status or health status. Few events are detected by a single method in every case, but the exercise yields insight into the relative usefulness of each detection method and the overlap of features between them.

**Figure 1:**
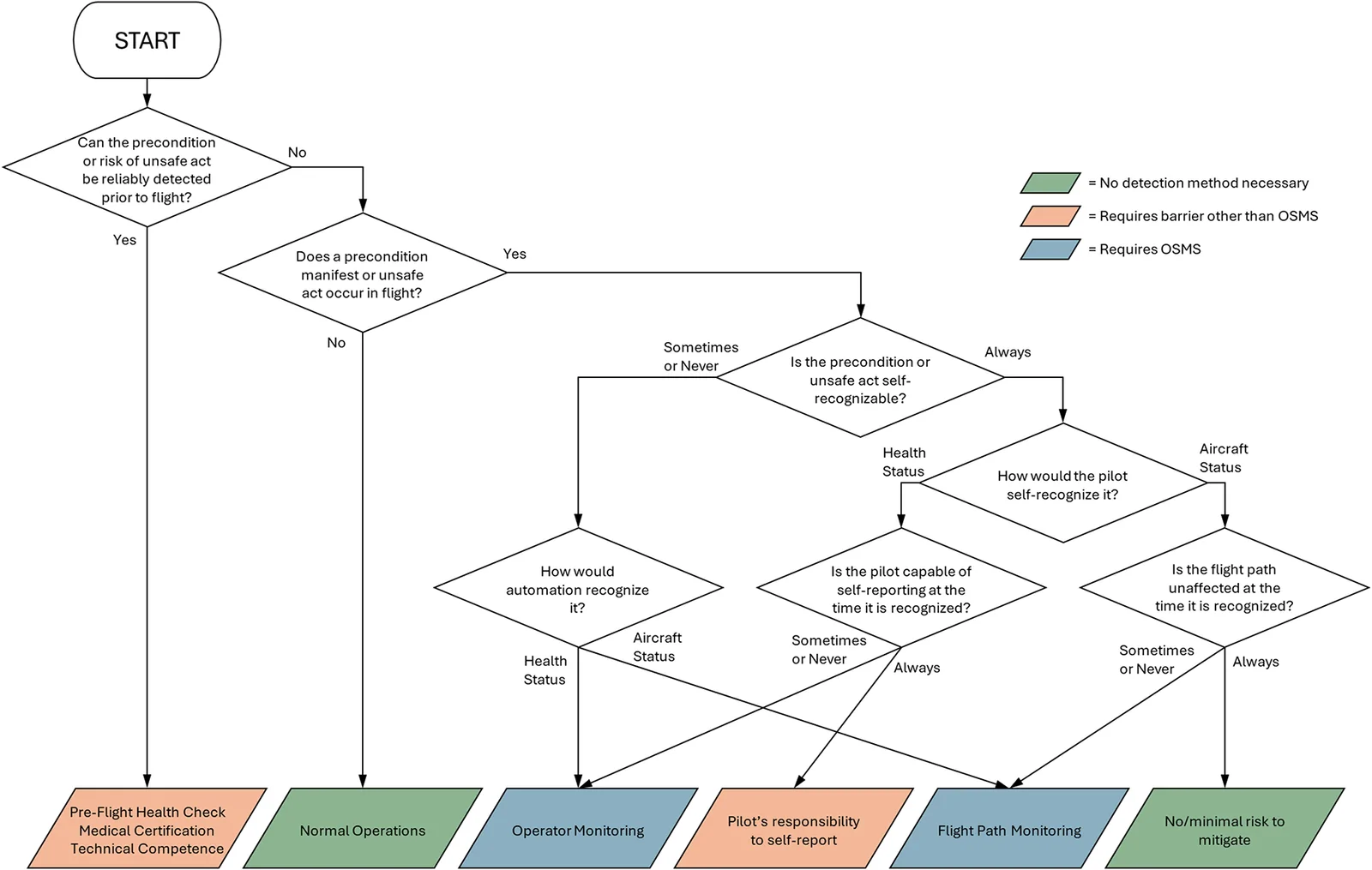
Detection method flow diagram for pilot impairment and incapacitation events.

Events are routed through questions about pre-flight detectability, in-flight manifestation, self-recognizability, and the primary manifestation pathway (health status vs. flight status) to identify candidate detection methods. The selected physiological events from section 2.3 were put through the flow diagram to determine potential detection methods.

#### 3.1.1 Self-report

Self-report is most reliable during the early and mid-stages of an impairment event, when the pilot retains the capacity and the situation awareness (SA) to recognize and communicate a degraded state. Pilots can self-report their condition using subjective assessment techniques and communicating their condition with their flight crew and/or with air traffic control (ATC). While the self-recognition of symptoms is an important contributor to flight safety, self-report as a stand-alone method is insufficient and may collapse when events become most dangerous. Hypoxia onset can be insidious, degrading judgment and self-assessment early, so a pilot’s ability to recognize their own state erodes before incapacitation. In a controlled hypoxia exposure, pilots did not report symptoms until roughly eight minutes after onset (Borden et al., 2024). Once impairment progresses towards incapacitation, the pilot may lose judgment, consciousness, or motor capacity to report. Thus, alternative and supplementary detection methods are necessary.

#### 3.1.2 Physiological/behavioral monitoring

Physiological monitoring exhibits the opposite severity profile. While self-report in Borden et al. (2024) did not occur until eight minutes into hypoxia exposure, includes arterial oxygen saturation (SpO_2_) dropped and HR increased within two to three minutes of onset, making it a faster and more sensitive indicator. It thus provides a detection window that neither self-report (the pilot may be unable to respond) nor flight path monitoring (the aircraft is still on course) can offer. Subtle degradations may not produce signatures distinguishable from normal individual variability, particularly for events with gradual onset; as an event progresses, however, characteristic changes become increasingly detectable and unambiguous.

To determine which signals an OSMS should monitor, a repository of pilot impairment and incapacitation events was created that identifies the affected physiological features. These physiological features are obtained by extracting certain characteristics from physiological signals (e.g., amplitude/timing of peaks, key points within an image). For example, changes in HR are associated with acute cardiac events (Selker et al., 1997), the onset of loss of consciousness (LOC) (Rinkel et al., 2024), and fatigue. Similarly, eye behavior features can provide insights into LOC (Rinkel et al., 2024) and fatigue (Stoddard, 2021; Road Safety Programs, n.d.). These features were organized into six groups: heart (HR, heart rate variability (HRV), and blood pressure); breathing (airflow, modulations, and ambient air quality); behavioral (control inputs, grip force, speech, face detection and posture); eye (saccade amplitude and velocity, blink rate and duration, percentage eye closure, pupil size, and gaze direction); skin (arterial oxygen saturation, skin conductance, tissue saturation index, and total hemoglobin); and brain (alpha, beta, and theta waves and event-related potentials). The mapping below links each event category introduced in the section 2.3 Summary of Selected Pilot State Degradation Events with measurable physiological and behavioral feature groups. The mapping captures features that could be affected by an event of that type rather than symptoms that will always be present, and derives from the symptom profiles cataloged above:

- Cardiac events affect heart features (HR changes, elevated blood pressure), breathing features (increased respiration), and behavioral features (slumped posture, speech change) (Goff et al., 1998).
- Fatigue presents across heart (decreased HR), breathing (slowed breathing rate), behavioral (slowed reaction time, slumped posture), eye (increased percentage eye closure (PERCLOS) and blink duration), and brain features (increased theta power, decreased beta) (Okamoto-Mizuno et al., 2008; Bendak and Rashid, 2020).
- Respiratory events appear in breathing features (decreased airflow, changes in breathing rate) (Awtry et al., 2006).
- Hypoxia appears in breathing (increased respiration rate), eye (rapid saccades, increased blink rate, decreased pupil reflex), skin (decreased (SpO_2_)), and behavioral features (erroneous flight control inputs) (van der Post et al., 2002; Cymerman et al., 2005; Stepanek et al., 2014).
- Spatial disorientation shows up in behavioral (erroneous flight inputs) and eye (scan patternscombined with flight state data) (Daiker et al., 2018; Spatial Disorientation | SKYbrary Aviation Safety, n.d.).
- Stress appears in heart features (increased HR, decreased HRV, high blood pressure) (Kim et al., 2018).

Across these events, heart, breathing, and behavioral features are affected most frequently, making them broad coverage candidates for OSMS. Breadth of coverage, however, is not the same as diagnostic value. A feature affected by many events is by definition weakly specific to any one of them, and elevated heart rate (which appears across cardiac events, hypoxia, stress, and spatial disorientation) is also a normal response to high workload and challenging phases of flight. Broad-coverage features are prone to false alarms when used in isolation, which is why they are treated here as candidates for corroborated detection rather than as independent triggers, and why threshold and sensor-agreement tuning is addressed in Recommendation 5.

Cardiac features such as HR and HRV are oft-cited target features for OSM development (Shaw and Harrell, 2023; Warfighter Performance Group, 2024) and have the most extensive monitoring-validation literature in aviation and adjacent operator domains. A systematic review of 29 studies for pilot mental workload reported substantial variability in study design and inconsistent results regarding HRV differences under varying workload levels (Wang et al., 2024); a parallel systematic review of 18 driver-fatigue studies reported HRV-based detection accuracies ranging from 44% to 100%, attributing the spread to differences in study design (Lu et al., 2022). Within aviation, HRV measured during flight distinguished fatigue levels in a cohort of 90 pilots, with a machine-learning classifier reaching approximately 89% accuracy from HRV and respiratory-rate features (Guo et al., 2025); HRV and ocular metrics jointly classified simulator-induced mental fatigue at 92% accuracy (Qin et al., 2021); and HRV tracked maneuver difficulty in commercial airline pilots in a certified simulator (Cao et al., 2019) and instrument-approach workload in fighter pilots (Mansikka et al., 2016). The inconsistency reported in both systematic reviews is a principal reason this work does not treat any cardiac feature as a standalone indicator.

Translating monitored features into a state classification requires decision logic. Features exceeding defined thresholds could be classified as impairment or incapacitation. For example, HR below 50 beats per minute (bpm) or above 130 bpm may indicate an impairment-level cardiac event, while HR below 30 bpm or above 200 bpm may indicate an incapacitation-level event. Given individual differences in resting HR and many other features, setting accurate absolute thresholds is difficult; the thresholds could also be established relative to a person’s baseline, such as monitoring for a change in HR of 30 bpm over a certain time window. The thresholds should also be dynamic, since phases of flight and off-nominal conditions produce physiological changes that are expected rather than abnormal: elevated HR during a poor-weather approach should not be flagged as an impairment. Combining features such as pose (e.g., slumped over) and eye tracking (e.g., prolonged period of eyes closed) with an abnormal HR could collectively indicate an incapacitated pilot. While specific threshold definitions are outside the scope of this work, the examples above illustrate the type of decision logic an OSMS algorithm would apply. The thresholds would require empirical validation and adaptive calibration for individual pilots in operational settings.

While the analysis above identifies the physiological features that must be monitored and the logic for interpreting them, it is insufficient for defining the optimal sensor implementation. Critically, it has no reference to the amplitude of the feature value change during each incapacitation event; a change in HR during an episode of spatial disorientation would be expected to be of lower amplitude than the change in HR due to atrial fibrillation or ventricular tachycardia. The specificity with which certain physiological features indicate certain incapacitation events is also not captured. Examples of this strong relationship include the use of pulse oximetry or cerebral oximetry (measuring skin features) to indicate hypoxia, electroencephalography (brainwave features) to detect seizures, or glucose monitoring for hypoglycemia.

One must consider the difficulty in obtaining an accurate measure of the features in an operational environment. The amount of signal processing, level and region of body/skin contact, and strength of the raw biosignal can vary greatly between features or within different methods of extracting the same feature. Cardiac features illustrate this well. Electrocardiography (ECG) is the gold standard method for obtaining HR and HRV features. However, traditional ECG requires the application of multiple adhesive electrodes to a person’s torso, which may be too slow to prepare, uncomfortable, or distracting for some operators. Alternative methods of acquiring ECG signals such as at the hand or ear (Heijden et al., 2024) offer a less obtrusive form factor but can have inconsistent skin contact or lower signal amplitudes. Photoplethysmography (PPG) can measure cardiac activity and requires only a single point of skin contact virtually anywhere on the body; however, it is highly susceptible to motion artifacts.

The optimal physiological sensors in an OSMS will vary based on the use case. At minimum, it is necessary to consider the types of impairment and incapacitation that must be detected, the acceptable level of sensitivity and specificity, and the risk of distraction or discomfort caused to the operators while the required sensors are in use. Most applications will also have some limits on integration such as existing body-worn equipment preventing sensor placement at certain positions or environmental factors like background noise, vibration, or ambient illumination that interfere with sensor readings. For both body-worn and remotely installed sensors, smaller devices will simplify integration, improve operator acceptance, and reduce motion artifacts.

Beyond sensor selection, flight deck monitoring faces limitations at every stage. Acquisition is constrained by motion and vibration artifact and by helmets, masks, and harnesses that limit placement and cover camera-based measurement, with head-worn sensing facing well-documented barriers to cockpit integration (Hamann et al., 2026). Interpretation is complicated by the overlap between high-workload and impairment signatures, which contributes to inconsistent HRV findings (Wang et al., 2024), by baselines that vary widely between pilots (Zeng et al., 2024) and drift within a flight, and by HRV methods that assume multi-minute windows while incapacitation alerting may require decisions in seconds. Cockpit noise affects electroencephalogram (EEG), ECG, and electrodermal activity (Shen et al., 2025), so environmental exposures must be modeled as covariates rather than background noise to be filtered out. Finally, labeled in-flight impairment data are nearly nonexistent, leaving most classifier results simulator-derived (Lu et al., 2022). These limitations do not argue against physiological monitoring, but against physiological monitoring in isolation, and they motivate the recommendations in Section 3.3.1.

#### 3.1.3 Task performance/flight path monitoring

Flight path and task-performance monitoring are typically the latest to manifest evidence of impairment, as there may be periods of time where pilots are not performing specific flight tasks. Deviations from expected flight paths are a significant indicator. When a pilot deviates from a planned route without a clear and communicated reason, it may suggest a loss of situational awareness or cognitive impairment. A delay or lack of response to ATC instructions or cockpit alerts can signal that a pilot is either not fully attentive or is experiencing some form of incapacitation. However, not all deviations are indicative of impairment; pilots may intentionally deviate during high-workload or safety-critical situations. Therefore, task-performance indicators must be interpreted within operational context and should rely on patterns rather than single deviations. Timing is a further constraint, during spatial disorientation the pilot’s first externally-observable sign is often an erroneous control input, by which point the event is already affecting the aircraft. By the time flight path deviations are large enough to detect with confidence, the precondition has already begun to develop into an unsafe act.

Monitoring of task performance may be more challenging to implement than monitoring of flight path or responses to stimuli, as the monitoring system would be required to have advance knowledge of what constitutes “correct” versus “incorrect” task execution. Establishing these definitions could be trivial for some tasks, but very difficult for others depending on task duration, number of steps, and situational constraints. Task execution errors include inputting incorrect settings on flight instruments, lack of compliance with standard operating procedures, or mistakes in communication. These errors may stem from a compromised physiological state, whether due to fatigue, medical issues, or other external factors impacting the pilot’s cognitive functions.

Detection may also be enhanced by aircraft and environmental monitoring, where OSMS complements rather than replaces existing detection pathways. Hypoxia can be caused by pressurization or oxygen-system failure; aircraft sensors may flag the underlying condition first, but OSMS establishes whether the pilot is still capable of the emergency descent the situation demands. Spatial disorientation is the inverse case: no aircraft-side sensor can detect it directly, and because the event is a mismatch between the pilot’s internal model and the aircraft’s actual state, a detection path could involve assessing the pilot’s control inputs in conjunction with their physiological data (Daiker et al., 2018). Engine/oil fume inhalation is not an operator state but an aircraft-system failure, most reliably identified through environmental sensing (air quality, bleed-air contamination) and the pilot’s recognition. OSMS would detect the downstream effects (e.g., tremor, loss of coordination, breathing irregularities) at later points in time. In each case, OSMS may contribute a unique perspective: an objective assessment of whether the pilot remains fit to recover normal operations.

### 3.2 Operator state monitoring system response

As OSMS detects changes of operator state, the system provides notification or alert to primary (e.g., pilot) and secondary operators (e.g., ATC, cabin crew). System responses are scoped to onboard recovery and adaptation, meaning actions available to the pilot and the aircraft’s automation. Secondary operators are informed so they can adjust their own tasks and coordinate a broader operational response, but the framework does not assume they can act directly on the pilot’s state. Supporting human-autonomy teaming scenarios in various aerospace operations may further require autonomous systems to anticipate potential degradations of human operators (Neubauer et al., 2020; Rauffet, 2023). These concepts remain exploratory and are not intended to imply specific operational commitments. Within that scope, this work explored OSMS response as operator state interventions and preventive actions. In this work, “interventions” is defined as an action made by the autonomy or the aircraft to support the operator based on their current state, while “preventive actions” are suggestions for an operator to perform based on their operator state. Depending on the mission design, these actions can be optional and not required for the operator to perform.

Failure, Mode, Effects, and Criticality Analysis (FMECA) is a widely used safety analysis technique for identifying failure points in system components. FMECA is one of the primary analysis used in the design of fault management subsystems as part of spacecraft system development. Based on a suite of safety and failure analysis, including FMECA, fault monitors and both local and system-level responses are designed and architected. This work adapts FMECA by treating pilot degradation as a ‘failure mode’ that triggers defined compensating system responses. We use this framing as an analytical convenience, not a claim that the pilot is a system component, because pilots can degrade gradually, recover, and vary between individuals.

A preliminary FMECA was conducted on this basis, carrying forward fatigue as a representative gradual-onset degradation. The outcomes support: a) selecting failure modes that warrant attention (some may not require action); b) determining which signals to monitor and establishing thresholds for triggering compensating responses; and c) defining the types of responses that can restore the system to its normal operating state. In the case of fatigue, a relevant checklist (e.g., fatigue suggested aids) and system response as intervention should be triggered. If recovered from the degraded state, the SA checklist should be presented to help the operator get up to speed with the mission and operation information missed while they were dealing with their degraded state. When SA is achieved, the mission tasks could be given back to the operator. If not recovered, the level of autonomy (LOA) of aircraft systems could be adapted for emergency response (**Figure 2**).

**Figure 2:**
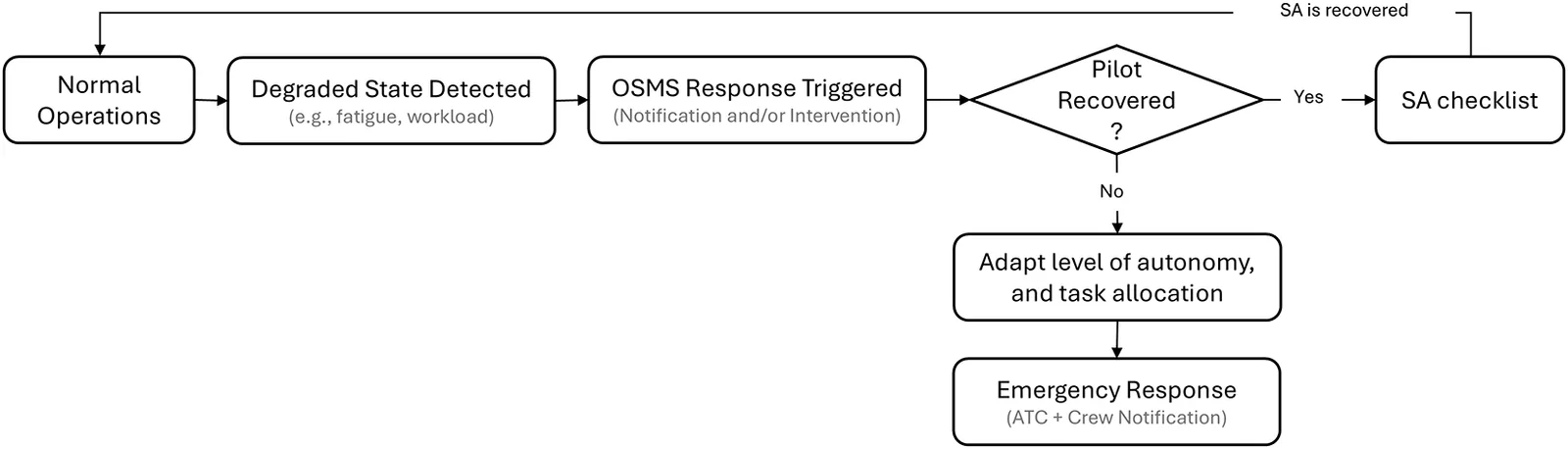
Simplified system response protocol.

### 3.3 OSMS design guidance and recommendations

#### 3.3.1 OSMS design recommendations

Industry systems already address part of this design space. Pilot Activity Monitoring (PAM) (IFALPA, 2014), for example, triggers the alerts when pilots fail to perform expected actions within a defined time window, providing a behavioral check on pilot engagement. Aircraft state monitoring approaches, similar to in-flight health monitoring systems (Airbus, 2022), can flag when control inputs become inconsistent with the aircraft’s current state, e.g., a pilot pressing forward on the controls while the aircraft is already nose-down. These existing systems are valuable for detecting consequences of pilot impairment, but they share a common limitation: they cannot predict or detect impending physiological events before performance degradation occurs. An OSMS designed around the multi-modal framework in Section 3.1 extends this existing capability by adding physiological and behavioral signal monitoring that can detect impairment earlier in its progression.

The recommendations below are derived from the prior systems and gaps reviewed in Section 2 and from the implementation limitations cataloged in Section 3.1.2. Recommendations 1 and 2 address what the system should monitor, extending coverage beyond the workload and fatigue focus of existing systems to the acute-event end of the impairment continuum. Recommendations 3 through 11 address how the sensing and processing must be engineered for the flight deck environment.

Recommendation 1: OSMS should be developed based on a multi-modal data approach consisting of physiological features, pilot performance and activity, aircraft state, and environmental data as well as a means to self-report input.

Recommendation 2: Pilots should have the ability to report their symptoms (or how they feel) by providing an input to the OSMS which can adapt its predictions based on the pilot’s self-report.

Recommendation 3: Sensors must be placed in a way that does not interfere with the pilot’s duties or comfort. If wearable sensors are employed, they need to be unobtrusive and durable, with solutions to operational challenges such as batteries requiring recharge or disposable components requiring restocking. In the cockpit specifically, sensors must not obscure instrument visibility, interfere with headset or oxygen mask use, or create discomfort during high-workload maneuvers such as emergency procedures. Note that the interpretation of unobtrusiveness may vary between user groups based on the equipment and procedures currently in place. For example, the application of adhesive electrodes to the torso for HR monitoring may be too cumbersome for commercial pilots but reasonable for a military application.

Recommendation 4: Sensors must provide accurate and reliable data in the dynamic aircraft environment. Factors like turbulence, varying cabin pressure, and pilot movements can create artifacts. Additionally, the monitoring system must be tested to verify reliability and accuracy. Redundancy mechanisms must be in place to maintain continuous monitoring and minimize the risk of system failure.

Recommendation 5: The system must minimize false positives (incorrectly identifying incapacitation) and false negatives (failing to detect a true incapacitation) to maintain an ALoS. Achieving this balance requires sophisticated data processing and validation techniques to output incapacitation alerts in a sufficient amount of time. The thresholds for duration, severity, and sensor-to-sensor level of agreement that are required to trigger an incapacitation alert/response must be tuned based on the application, as the relative appetite for false positives versus false negatives will vary based on the implications of each in each operational environment. In aviation, the cost of both error types is especially high: a false positive that triggers an unnecessary emergency response can itself create a safety hazard, while a false negative during a critical flight phase can result in safety hazards and, in extreme cases, be fatal.

Recommendation 6: Multiple sensing modalities should be used to capture different aspects of a human’s health and behavior. The data from each sensor must be synchronized accurately for a successful OSMS, as any time lags or misalignments can lead to incorrect assessments of the pilot’s condition. Data will need to be aggregated and analyzed from multiple sources including physiological signals, flight state data, and self-reports.

Recommendation 7: OSMS design should consider how to maintain monitoring continuity during flight, recognizing that full redundancy may not always be feasible within existing aircraft power architectures. Interruptions can compromise the monitoring system’s effectiveness.

Recommendation 8: Cross-sensor interactions must be mitigated. Sensors may influence each other’s measurements, especially in the confined space of an aircraft cockpit, through shared sensing modalities or by inducing a change in the underlying physiological signals. For example, there may be interactions of the infrared light used for camera-based monitoring and pulse oximetry, or modified posture or breathing caused by a contact-based sensor affixed to the torso.

Recommendation 9: The system must process and analyze data in real-time to provide timely alerts during the operations. This requires robust computational resources and efficient algorithms. Alert latency requirements in aviation are more stringent than most other domains – a delayed incapacitation alert during approach or takeoff may provide insufficient time for automated or ATC intervention. Latency requirements for a specific use case should be determined based on the total time required to address the safety hazard caused by an adverse operator state, including both the time for detection and the subsequent system response (e.g. a responsive aircraft maneuver).

Recommendation 10: Each sensor must be calibrated correctly to ensure consistent and accurate reading. Pilot-to-pilot physiological variability, combined with the impracticality of extended baseline collection periods before each flight, makes adaptive per-pilot calibration a distinct challenge for aviation deployment.

Recommendation 11: Airworthiness qualification must be treated as a foundational design driver, not a downstream detail. Any sensing hardware integrated within aircrew flight equipment for the flight deck is subject to airworthiness qualification, which imposes environmental, electrical-safety, materials, oxygen-compatibility, electromagnetic-compatibility, and mechanical-integrity requirements that constrain sensor form factor, power architecture, and integration. This is primarily for the case of commercial and military operational implementations; this does not apply to training and simulation environments.

#### 3.3.2 Design guidance: operator state monitoring system interface for pilots

The OSMS for pilots minimally includes an interface to communicate the information with the operator themselves; there may also be secondary operators such as cabin crew, ATC, or a ground station operator for flight operations. Human-Machine Interface (HMI) design should consist of adaptable interface and interaction artifacts that enable OSMS to be reconfigurable for various concepts of operations. Major principles for an OSMS HMI include:

- *minimal distraction* when the pilot does not need to pay attention to their states,
- *transparency* by explaining how confident the detection system is,
- *explainability* by providing options for pilot or secondary human agents to review the detailed construct list for causality of the pilot state notification,
- *controllability* by providing options for a pilot to acknowledge the state or manually escalate the alert mechanism for suitable system response as well as override when needed.

Both Human Factors Design Guidance for Driver-Vehicle Interfaces (Campbell et al., 2016), and Pilot Medical Monitoring: State of the Science Review on Identification of Pilot Incapacitation (Brokaw et al., 2022) were used as references to derive the guidance for HMI design of an OSMS. There are five main areas for the alerts and notification system requirements that influence HMI design:

1. System Design Principles
  - Reliability: account for potential failures and maintain consistent operation
  - Timing: issue alerts with sufficient time for appropriate operator response
  - Operator Focus: avoid overloading or distracting the operator
  - Customization: enable integration into various platforms with voice and touch interfaces
2. Alert Classification and Escalation
  - Severity Levels: implement four escalation levels (Advisory, Caution, Warning, Incapacitation/Emergency declaration) that map to the pilot states, where advisory and caution map to developing impairment, warning to significant impairment, and incapacitation/emergency to full incapacitation
  - Distinct Patterns: use unique alert patterns for each severity level and message type
  - Intensity Mapping: adjust alert intensity to match the escalation levels
  - Language: use clear, unambiguous instructions that support situational awareness without overloading the operator
3. Multi-Modal Alerts
  - Visual Alerts
    - Present concise messages within operator’s primary line of sight
    - Use pictograms/symbols for time-critical situations
    - Use text for comprehension-critical situations
  - Auditory alerts
    - Provide quick attention-drawing signals
    - Use distinct tones that are not already in the cockpit
  - Haptic alerts: use distinct seat vibrations for highly critical states when visual and auditory channels may already be saturated during an emergency.
4. Status Display and Feedback
  - Continuous status: display the pilot state throughout operations (Nominal = green, Impaired = yellow, incapacitated = red)
  - Recovery feedback: provide confirmation when recovery is achieved
  - Explanatory information: present operator state and potential cause
  - Confidence indicators: show confidence levels of OSM detection
5. Operator Control and Guidance
  - Override capability: allow operator to override false positive alerts
  - Preventive actions: suggest specific actions to support pilot recovery
  - Intervention options: allow the operator to request the system intervention (e.g., autonomy takeover).

## 4 Discussion

This work suggests that OSMSs should not be used to diagnose specific impairment and incapacitation events. Instead, it must detect and categorize the operator’s state changes as normal, impaired, and incapacitated. Combining multiple detection methods can result in improved categorization and detection of pilot state to further take action to prevent any flight risk.

Even with a multi-modal approach, there will be coverage limitations to detect a variety of conditions. A pilot who has lost consciousness has no reliable self-report path and may not produce flight path deviations during autopilot-assisted flight, leaving physiological monitoring as the sole detector. Spatial disorientation offers no pre-flight detection and no self-report pathway, since a disoriented pilot by definition does not recognize their disorientation. These gaps identify priority targets for future sensor development and algorithm research, and they reinforce the postulation that no single detection method is sufficient for the full range of in-flight impairment and incapacitation events.

These research activities also included FMECA for understanding the failures and system responses as a result of pilot degradation (identified as failure causality). This effort demonstrated the value of treating the human operator as part of the overall system design and which identified failures/errors could be mitigated through system response design. Further development of FMECA and its investigation are needed to test and validate the approach with adaptive automation response as mitigation method.

Pilot acceptance is a distinct challenge for OSMS deployment. Resistance to new technology can be a significant barrier, and training on benefits of the system and communication may help build pilot acceptance over time. Acceptance will also be greatly influenced by what data is collected, how long it is retained, and who has access under which conditions, so privacy concerns around data handling and protection must be addressed and clearly communicated with operators as the technology develops.

While the focus of this research was to identify an OSMS framework, the next potential steps are to build the prototype system and empirically test for validation in operationally relevant environments. Additionally, iteration based on the testing and further analysis should be performed on the risk and failure assessment with potential mitigation methods and mitigation techniques as system response.
